# Juvenile Hormone and Ecdysteroids Facilitate the Adult Reproduction Through the *Methoprene-Tolerant* Gene and *Ecdysone Receptor* Gene in the Female *Spodoptera frugiperda*

**DOI:** 10.3390/ijms26051914

**Published:** 2025-02-23

**Authors:** Yan Zhang, Kui-Ting Ding, Nian-Wan Yang, Zhi-Chuang Lv, Zhen-Ying Wang, Yong-Jun Zhang, Wan-Xue Liu, Jian-Yang Guo

**Affiliations:** 1 State Key Laboratory for Biology of Plant Diseases and Insect Pests, Institute of Plant Protection, Chinese Academy of Agricultural Sciences, Beijing 100193, China; 17810266106@163.com (Y.Z.); 18198261029@163.com (K.-T.D.); yangnianwan@caas.cn (N.-W.Y.); lvzhichuang@caas.cn (Z.-C.L.); wangzhenying@caas.cn (Z.-Y.W.); zhangyongjun@caas.cn (Y.-J.Z.); 2 State Key Laboratory of Resource Insects, Institute of Apicultural Research, Chinese Academy of Agricultural Sciences, Beijing 100193, China

**Keywords:** *Spodoptera frugiperda*, hormone, reproduction, *Met*, *EcR*, RNA interference

## Abstract

Insects, as the most diverse and numerous group in the animal kingdom, are at least partly dependent on the reproduction process, which is strictly regulated by the ‘classic’ insect hormones: juvenile hormone (JH), and 20-hydroxyecdysone (20E). However, the regulatory mechanism governing the reproduction of JH and 20E in *Spodoptera frugiperda* remains unclear. In this study, ovarian development and ovulation in female *S. frugiperda* were assessed through dissection of the ovaries following treatment with JH analog (JHA) and 20E. Moreover, the expression patterns of the JH-signal and 20E-signal-related genes were determined by quantitative PCR (qPCR), and RNA interference (RNAi) was used to investigate the role of JH and 20E-induced genes. Ovarian development was observed by microdissection, and JH and 20E titers were determined by ELISA. *Kr-h1*, *Vg*, and *USP* expression were determined by qPCR. Dissection and qPCR results showed that JHA and 20E promoted ovarian development, egg maturation, and egg laying by upregulating *Methoprene-Tolerant* (*Met*) and *Ecdysone Receptor* (*EcR*)expression. Additionally, the RNAi results showed that the injection of ds*Met* and ds*EcR* markedly delayed ovarian development, inhibited egg maturation, and halted egg production. Knockdown of *Met* and *EcR* significantly reduced JH and 20E content and inhibited the transcription of *Kr-h1* and *USP*. These results indicate that JH and 20E facilitate adult reproduction through the methoprene-tolerant gene and ecdysone receptor gene in female *S. frugiperda.*

## 1. Introduction

Sexual reproduction requires the involvement of sexual endocrine hormones. Endocrinology of insect reproduction has traditionally focused on juvenile hormones (JHs) and ecdysteroids, which regulate development and reproduction and are well studied in most important insect groups, such as Lepidoptera, Coleoptera, and Diptera [1,2]. The proper synthesis and functioning of JHs and ecdysteroids are crucial for regulating vitellogenesis and oogenesis, from embryo development to the production and laying of fertile eggs [3,4].

JHs are lipophilic sesquiterpenoid molecules secreted by the corpora allata (CA), a pair of endocrine glands located just behind the brain [5]. Eight distinct types of JH have been discovered in various insects, with JH III being predominant. Vitellogenesis is regulated by the release of JHs, which travel through the hemolymph and target tissues with specific receptors. This triggers the biosynthesis of vitellogenin in the female fat body and follicle cells, along with the absorption of yolk protein precursors by oocytes in developing follicles [4,6]. In addition to its role as a regulator of reproduction, ecdysteroids, as multifunctional hormones, also play a significant role in the adult stage of many insects [7]. Ecdysteroids are polyhydroxylated sterolic hormones [8]. Generally, the prothoracic glands produce ecdysteroids in response to stimulation by prothoracicotropic hormone (PTTH) and release them into the hemolymph [9]. Ecdysteroids produced in the ovaries are stored in oocytes for use during ovary development, oocyte maturation, and embryogenesis [2,10].

Recent years have witnessed an explosion of studies on JHs and ecdysteroid signal pathways [6,7,11]. Although the regulation of vitellogenin expression and ovarian development by JH has been reported for many years, the molecular mechanisms gained attention with the discovery that the JH-resistance gene, *Methoprene-tolerant* (*Met*), a basic helix-loop-helix Per/Arnt/Sim (bHLH-PAS) domain protein, acts as the JH receptor, binding JH at physiological levels, and playing a key role in insect metamorphosis [1,12,13]. However, the pathway of action on ecdysone depends on 20-hydroxyecdysone (20E), the active form of ecdysteroids. The transcriptional activation of target genes is triggered by 20E, as it forms a nuclear receptor complex with the Ecdysone receptor (EcR) and its partner, the orphan nuclear receptor Ultraspiracle (USP). The 20E regulatory pathway is essential for vitellogenesis and oogenesis in dipterans [1].

The fall armyworm (FAW), *Spodoptera frugiperda* (Lepidoptera: Noctuidae), is a major invasive pest affecting a range of crops, especially maize and other cereals in its invasion area, such as North and South America [14]. By early December 2018, it had also spread from Myanmar into Yunnan Province in southern China [15].

Given the high fecundity, migration capabilities, and population resistance to insecticides (e.g., pyrethroids, carbamates, and organophosphates) of FAW, pest control strategies are limited [16]. In the polyandric moth, oogenesis is completed only after adult emergence, and oviposition closely depends on endocrine hormones (JH and 20E) [17]. However, as far as we know, the roles endocrine hormones play in FAW’s reproduction, and whether JH-Met signaling and 20-EcR regulate the reproductive process, are still unknown. To address these questions, we use two important hormones secreted by glandular secretory organs as a model to study how JH and 20E function to promote high fertility in *S. frugiperda.* Specifically, we focus on two key receptors of the JH and 20E signal pathways and set out to clarify whether egg maturation and oviposition are modulated by the receptors and cascade of genes. We employ anatomical analysis, ELISA, qPCR, and RNAi to examine, characterize, and identify the mechanisms through which the hormones regulate reproductive processes in female adults. We show that exogenous administration of juvenile hormone analog (JHA) and an ecdysteroid (20E) can promote egg maturation and oviposition, in which *Met* and *EcR* play key roles as hormone receptors. Furthermore, the results show that *Met* and *EcR* regulate hormone synthesis and cascade gene expression. Our results highlight the crucial role of JH and ecdysteroids in facilitating adult reproduction through the *Methoprene-tolerant gene* and ecdysone receptor gene in female *S. frugiperda.*

## 2. Results

### 2.1. JHA and 20E Induce Ovarian Development and Egg Laying

**Figure 1:**
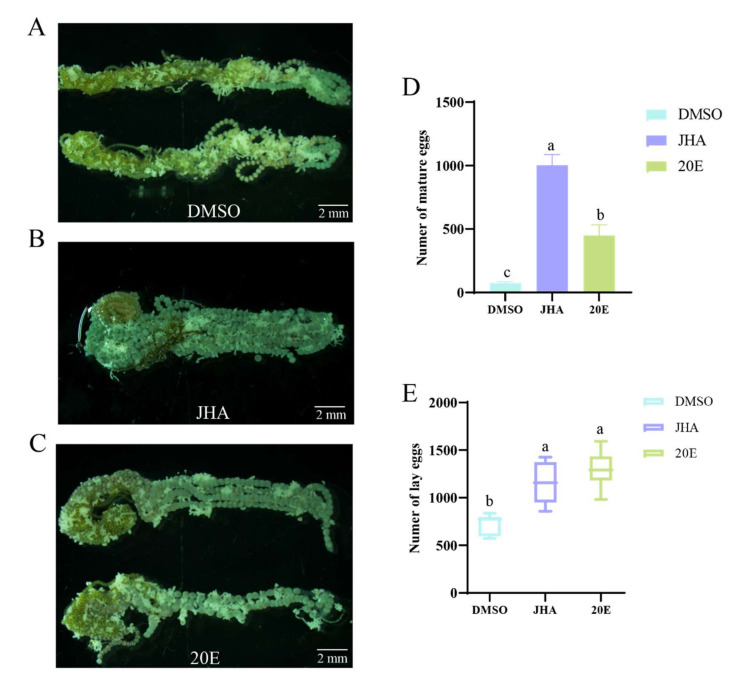
JHA and 20E promotes ovarian development and egg laying. (**A**) Ovary of *S. frugiperda* after DMSO injection; (**B**) Ovary of *S. frugiperda* after JHA injection; (**C**) Ovary of *S. frugiperda* after 20E injection; (**D**) Number of mature eggs of *S. frugiperda* after injection of DMSO, 20E and JHA; (**E**) Number of laid eggs of *S. frugiperda* after injection of DMSO, 20E and JHA. Mature eggs and laid egg counts presented as the Means ± SE. The data analysis was conducted using a one-way ANOVA, followed by the LSD test (columns labeled with different lowercase letters differ significantly from one another, *p* < 0.05).

To explore the effects of a juvenile hormone (JH) and 20-hydroxyecdysoen (20E) on ovarian development and egg production, we initially assessed the potential of exogenous JHA and 20E to promote ovarian development in *S. frugiperda*. We treated newly emerged females (without feeding) with JHA and 20E, and dissected the ovaries at 24 h post-injection. Compared with the DMSO control, both 2.5 µg JHA and 5 µg 20E promoted ovarian growth (Figure 1A–C). Moreover, the number of mature eggs in ovaries was 3010 for JHA-treated females and 1347 for 20E-treated females (Figure 1D), whereas in the DMSO-treated females, it was 222 (Figure 1D). Therefore, JHA and 20E induce the maturation of eggs in the ovaries (Figure 1D). Furthermore, we investigated egg laying in females. The results showed that DMSO-treated control females laid 7209 eggs, while JHA-treated females and 20E-treated females laid 11,496 and 13,002 eggs, respectively (Figure 1E). Therefore, JHA and 20E induced ovarian development, accelerated the rate of oocyte maturation, and increased egg laying. These results suggest that JH and 20E signaling contribute to the facilitation of reproduction in *S. frugiperda*.

### 2.2. JHA and 20E Upregulate the Levels of the JH and 20E Pathway-Related Gene Transcripts

**Figure 2:**
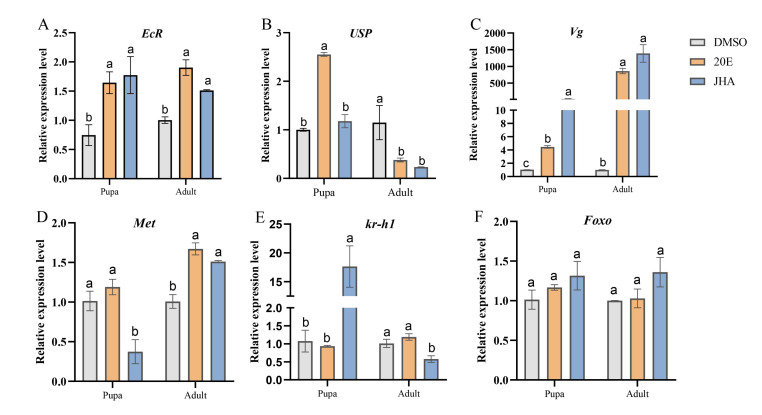
Injection of DMSO, 20E and JHA into female of *S. frugiperda* upregulates JH and 20E signal pathway relative gene expression level. (**A**) *EcR*; (**B**) *USP*; (**C**) *Vg*; (**D**) *Met*; (**E**) *Kr-h1*; (**F**) *FOXO*. All values are shown as Means ± SE. Columns labeled with different lowercase letters differ significantly from one another, *p* < 0.05. The data analysis was conducted using a one-way ANOVA, followed by the LSD test.

In insects, ecdysteroid signaling is mediated by the heterodimer of *EcR* and *USP* nuclear receptors, while JH exerts its effects through the *Met* receptor, initiating the transcription of downstream targets such as *Kruppel homolog 1* (*Kr-h1*) and *Foxo*. To verify the relationship between reproduction and JH and 20E pathway-related gene expression, we tested the mRNA expression levels of *EcR*, *USP*, *Met*, *Vg*, *Kr-h1*, and *Foxo* in pupae and females after JHA and 20E treatment using qRT-PCR. Clearly, compared to the DMSO treatment group, after a 24-h induction with JHA or 20E, the expression of these genes was upregulated in response to JHA and 20E in pupae and reproductive females (Figure 2A–F), but *Foxo* exhibited no significant change for JH or 20E (Figure 2F). EcR mRNA levels increased by 1.51-fold in response to JH signaling and 1.90-fold in response to 20E signaling in female adults (Figure 2A). In the same situation, *Met* mRNA levels increased 1.50-fold and 1.70-fold, respectively (Figure 2D). Especially, after JHA and 20E treatment, Vg expression increased over 1387-fold and 859-fold, respectively (Figure 2C). The findings indicated that the expression of *EcR*, *Met*, and *Vg* genes is enhanced in response to JH and 20E signaling during adult reproduction.

### 2.3. EcR and Met Expression in Different Stages of Development and in Various Tissues

**Figure 3:**
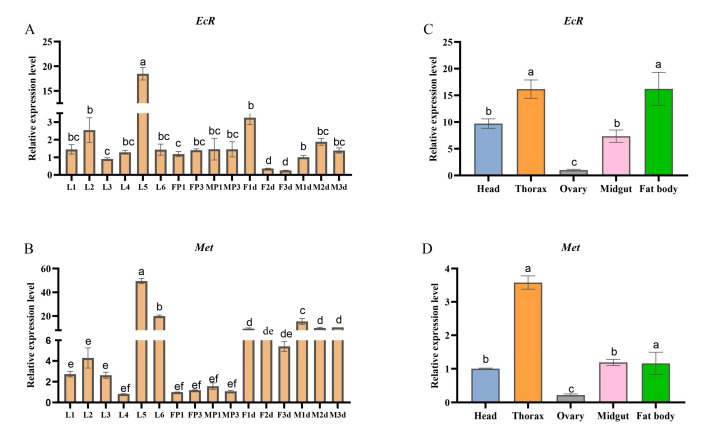
*EcR* and *Met* expression at different developmental stages (**A**,**B**) and tissues (**C**,**D**). The value are expressed as the Means ± SE. Columns labeled with different lowercase letters differ significantly from one another, *p* < 0.05), the data analysis was conducted using a one-way ANOVA, followed by the LSD test.

To characterize the *EcR* and *Met* expression profiles in different stages of development and in various tissues, qRT-PCR was used to detect the expression levels of *EcR* and *Met* for larvae (L1–L6), pupae (FP, MP), and adults (F, M). During the *S. frugiperda* developmental process, *EcR* gene expression was 18-fold higher at the end of the larval stage (L5) than at the pre-larval stage (L3), and there was no difference between the expression at the pupal stage and the adult stage (Figure 3A). Similarly, a higher expression level of *Met* genes was observed in the fifth larval stage, while the expression levels in the L1–L4 stages and the pupal stages were not very different, and in the adult stage, *Met* was significantly higher than that in the larval stage and pupal stage (Figure 3B). For the different tissues, the results showed that EcR was more highly expressed in the thorax and fat body than in the head, ovary, and midgut (Figure 3C), whereas *Met* levels were highest in the thorax (Figure 3D). These results imply that *EcR* and *Met* are likely involved in key roles in specific developmental processes and endocrine activity.

### 2.4. Knockdown of Met and EcR Results in Ovarian Development Retardation and Decreased Egg Laying

**Figure 4:**
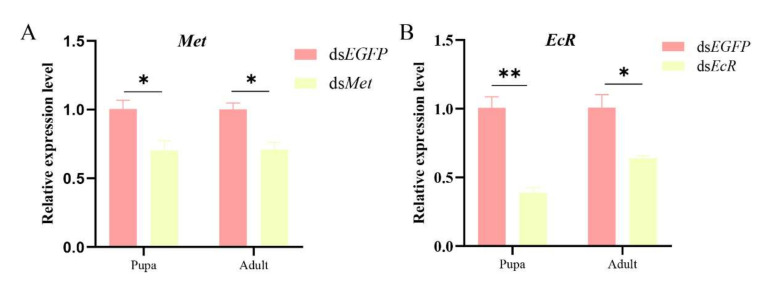
Relative expression of *Met* and *EcR* after ds*Met* and ds*EcR* injection of *S. frugiperda.* (**A**) *Met* gene relative expression level; (**B**) *EcR* gene relative expression level. The values are presented with the Means ± SE. The asterisks indicate the significance level, as determined by one-way ANOVA followed by the LSD test. *, *p* < 0.05; **, *p* < 0.01.

**Figure 5:**
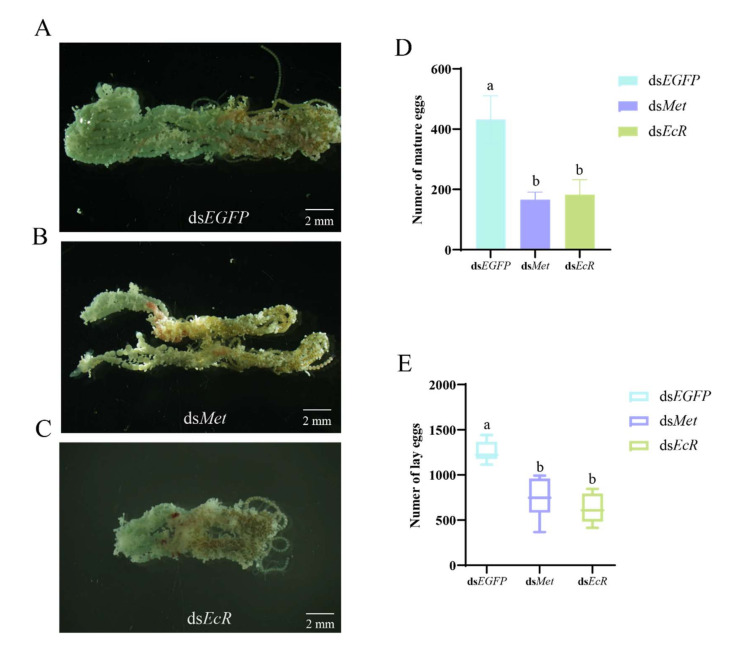
*Met* and *EcR* affect ovarian development retardation and decreased egg laying. (**A**) Ovary of *S. frugiperda* after ds*EGFP* injection; (**B**) Ovary of *S. frugiperda* after ds*Met* injection; (**C**) Ovary of *S. frugiperda* after ds*EcR* injection; (**D**) Number of mature eggs of *S. frugiperda* after injection of ds*EGFP*, ds*Met* and ds*EcR*; (**E**) Number of laid eggs of *S. frugiperda* after injection of ds*EGFP*, ds*Met* and ds*EcR*. The number of mature eggs and laid eggs are expressed as the Mean ± SEM. The statistical analysis was performed using a one-way ANOVA followed by the LSD test (columns with different lowercase letters are significantly different from each other, *p* < 0.05).

To clarify the functions of *Met* and *EcR* of *S. frugiperda*, two key receptor genes of the JH and 20E hormone pathway, we performed RNAi to investigate the effect of *Met* and *EcR* on JH-/20E-induced ovarian development. One-day-old female pupae and female adults were injected with ds*EcR* and ds*Met*. Compared with the ds*EGFP* control, ds*Met* injection caused a 29% reduction in expression in female pupae and adults (Figure 4A), while ds*EcR* injection reduced expression in pupae and adults by 61% and 36%, respectively (Figure 4B). By dissecting the ovaries of females, compared with the ds*EGFP* group (Figure 5A), significantly less yolk protein was deposited in the ovaries of the ds*Met*-RNAi (Figure 5B) and ds*EcR*- RNAi groups (Figure 5C). Furthermore, it was found that the number of mature eggs in the ovaries of *Met* -RNAi females was reduced by 41% (Figure 5D), and the number of laid eggs by females was reduced by 42% (Figure 5E). Similarly, *EcR*-RNAi females had a significant 42% reduction in mature eggs in the ovaries and a 49% reduction in female egg production (Figure 5D,E). These results strongly demonstrate that silencing *Met* and *EcR* affects yolk deposition, egg maturation, and oviposition in *S. frugiperda*.

### 2.5. Knockdown of Met and EcR Results in Decreased JH, 20E Titer and Vg, Kr-h1 and USP Expression Levels

**Figure 6:**
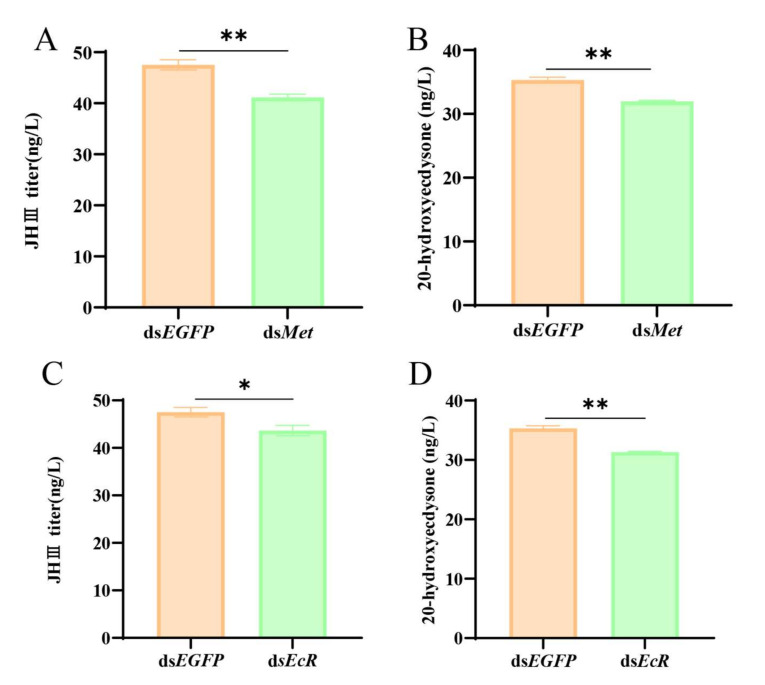
Effects of *Met* and *EcR* on JH and 20E. (**A**) and (**B**) respectively represent the titer of JH and 20E after ds*Met.* (**C**) and (**D**) respectively represent the amount of JH and 20E after ds*EcR*. The titer levels are expressed as the Means ± SE. The data analysis was conducted using a one-way ANOVA, followed by the LSD test (*p* < 0.05; * and ** represent *p* < 0.05 and *p* < 0.01, respectively).

**Figure 7:**
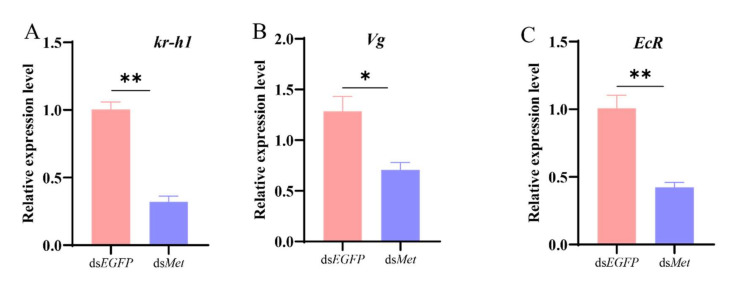
Effects of ds*Met* injection on *Kr-h1*, *Vg and EcR* expression levels. (**A**) *Kr-h1* gene; (**B**) Vg gene; (**C**) *EcR* gene. The mRNA levels are expressed as the Means ± SE. The data analysis was conducted using a one-way ANOVA, followed by the LSD test (*p* < 0.05; * and ** represent *p* < 0.05 and *p* < 0.01, respectively).

**Figure 8:**
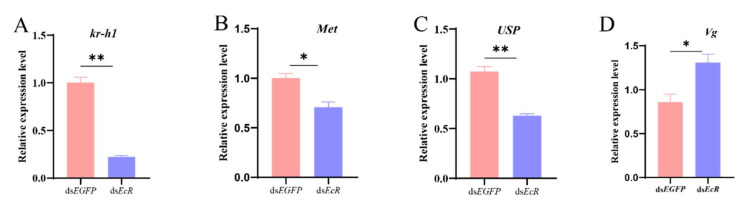
Impact of ds*Met* injection on *Kr-h1*, *Met*, *Vg and EcR* expression levels. (**A**) *Kr-h1* gene; (**B**) *Met* gene; (**C**) *USP* gene; (**D**) *Vg* gene. The mRNA levels are expressed as the Means ± SE. The data analysis was conducted using a one-way ANOVA, followed by the LSD test (*p* < 0.05; * and ** represent *p* < 0.05 and *p* < 0.01, respectively).

*Met* and E*cR*, as the vital receptors, are closely related to JH and 20E synthesis. To further confirm the direct relationship of *Met* and *EcR* with the hormones, after silencing the *Met* expression and *EcR* in female adults, the JH and 20E titers were determined. Significant JH and 20E titer decreases were observed in the experimental groups, the ds*Met* -RNAi group (Figure 6A,B), and the ds*EcR*- RNAi group (Figure 6C,D), relative to the control group. Furthermore, the previous results also proved that *Vg* [18] and *Kr-h1* [19] are crucial for the reproduction of *S. frugiperda*. Therefore, after ds*Met* injection, the data indicated a 68%, 45%, and 58% decrease in the transcription levels of *kr-h1*, *Vg*, and *EcR*, respectively, relative to the control group (Figure 7A–C). Meanwhile, after ds*EcR* injection, the relative expression level of *kr-h1* and *Met* transcript levels were reduced by 88% and 29%, respectively, and the *EcR* heterodimerization partner (the *USP* expression level) was also reduced by 41% (Figure 8A–C). However, knocking down the *EcR* resulted in a 1.5-fold increase in the expression level of *Vg* (Figure 8D). *Met* and *EcR* affect the hormones via the mRNA expression level of pathway-associated genes.

## 3. Discussion

Reproduction in insects is regulated by the hormones ecdysone and juvenile hormones and the availability of nutrients [20,21]. Understanding insect reproduction processes can support the development of new strategies for pest control [22]. Native to America, *S. frugiperda* is a destructive pest that has recently invaded China, with its prolific reproduction being a key driver of its devastating impact [16]. However, the mechanisms of JHs and ecdysone in controlling its reproductive processes are not yet well understood. In the current research, we analyzed the roles of JHA and 20E in the reproductive processes of *S. frugiperda*, cloned and analyzed key genes (*EcR*, *USP*, *Vg*, *Met*, *Kr-h1*, and *Foxo*) of the JH and 20E signaling pathways, and analyzed the induction of JHA and 20E on their mRNA expression levels. Then, we identified two key receptor genes, *Met* and *EcR*, of the JH and 20E signaling pathways induced by JHA and 20E, and assessed their role in female adult reproduction through RNA interference.

The development and reproduction of different insect taxa may be regulated by a single or multiple factors [23]. JH- and 20E-mediated larval development models have been commonly reported in many insects [6,7,24]. Furthermore, JH levels seem to be pivotal in regulating reproductive diapause, aging, migration of butterflies, and caste differentiation processes [25]. Recently, more studies have paid attention to JH- and 20E-mediated regulation of reproduction. For *S. frugiperda*, there is no stagnation of fertilization, and both females and males can mate several times and are extremely fertile. Therefore, it is a good model to study JH- and 20E-mediated regulation of reproduction. Similar to previous reports in *Colaphellus bowringi* [24], after injecting 2.5 µg JHA into females of *S. frugiperda*, ovarian development was induced, resulting in egg maturation and increased egg laying. As for the multifunctional ecdysteroids produced in the ovaries, Lanot et al. [26] proved that ecdysone is involved in regulating meiotic reinitiation in the oocytes of *Locusta migratoria*; mosquito ecdysone is also important in development and reproduction [27]. Recently, a study by Wang et al. [28] found that 20E influences many cellular processes in *S. frugiperda*. Similarly, when we applied 5 µg of 20E to *S. frugiperda* female adults, yolk accumulation in the ovaries increased, oocyte maturation was induced, and the number of laid eggs increased.

JHA methoprene and 20E achieve reproductive regulation depending on the related gene expression [29,30]. Typically, JHA induces *Vg* expression in *C. bowringi* [24], and *Kr-h1* gene expression is significantly increased by both JH analog (JHA) and 20E in *Helicoverpa armigera* [31]. Our study revealed that *Met* and *EcR*, key receptors in the JH and 20E signaling pathways, showed elevated expression following JHA and 20E treatment, suggesting their crucial role in hormone response, particularly in the thorax and fat body. Moreover, to further demonstrate whether JHA and 20E functionally involve the regulation of reproduction via *Met* and *EcR*. RNAi-based approaches have been effectively utilized to study gene function in different insect species, including Lepidoptera. In the present study, the *Met* and *EcR* genes of *S. frugiperda* were silenced, and *Met* and *EcR* expression was markedly downregulated in female pupae and adults after injection of dsRNA. Suppression of *Met* and *EcR* in *S. frugiperda* females impaired ovarian development and oogenesis, leading to an inhibition of egg maturation and reduced oviposition, and reduced the JH and 20E level. In addition, silencing the *Met* gene markedly downregulated *kr-h1*, *Vg*, and *EcR* expression levels. Additionally, knocking down *EcR* resulted in a significant reduction of *kr-h1*, *Met*, and *USP* expression, while *Vg* levels were higher than in the control ds*EGFP* group. Similarly, the *Vg* expression level also decreased after *EcR* expression increased in *Diploptera punctata* [32], which may be due to other genes of the 20E-signal pathway compensating for the *Vg* gene expression.

In line with previous research, our data suggest that *Met* and *EcR* affect reproduction in *S. frugiperda*. Earlier studies have reported that silencing *Met* expression through dsRNA injection in *Grapholita molesta* resulted in prolonged pupal duration, hindered adult emergence, prolonged the preoviposition period, and decreased fecundity [33]. Lenaerts et al. [34] discovered that silencing *EcR* in *Schistocerca gregaria* resulted in impaired ovulation and oviposition. Choi et al. [35] found that the development and reproductive capacity of *S. frugiperda* populations from the USA and China were influenced by ecdysone-related genes. Moreover, Guo et al. [36] proved that 20E controls photoperiodic reproductive arrest by modulating JH production, which is related to the gene cascade of JH-signal and 20E-signal pathways, such as *kr-h1*, *USP*, and *Vg* [21,37]. Importantly, the cross-talk between 20E and JH signaling pathways is mediated by the participation of calponin [29] and unknown key proteins. In summary, this study presents the possibility of preventing the developmental and reproductive cycles of *S. frugiperda* by inhibiting two critical receptor genes, providing a means to eliminate the damaging effect.

## 4. Materials and Methods

### 4.1. Insect Rearing

*Spodoptera frugiperda* specimens were collected in Nanning, Guangxi province. Colonies were established in the laboratory at the Institute of Plant Protection, Chinese Academy of Agricultural Science under controlled conditions of 25 ± 2 °C, 55 ± 5% RH, and a 14:10 h light: dark photoperiod for more than 10 generations. Larvae were raised in cylindrical feeding boxes (top cover length 4.0 cm, height 3.0 cm, bottom length 3.0 cm) using artificial feed (Corn flour-60%, Wheat germ powder-15%, Soybean flour-15%, Yeast powder-5%, Fish meal-3%, Agar-2%), while adults were raised in cuboid feeding cages (55 cm × 55 cm × 55 cm) with 20% honey water.

### 4.2. Collection of Sample for Developmental Stages and Tissue Expression Analysis

Samples from various developmental stages and sexes were collected to analyze the expression levels of candidate genes, including the first to sixth instar (L1, L2, L3, L4, L5, L6), first-day female pupa (FP1), third-day female pupa (FP3), first-day male pupa (MP1), third-day male pupa (MP1), 1-day-old female (F1d), 2-day-old female (F2d), 3-day-old female (F3d), 1-day-old male (M1d), 2-day-old male (M2d), and 3-day-old male (M3d). For tissue-specific analysis, the head, thorax, ovary, midgut, and fat body were dissected from female adults using a SZX16 microscope (Olympus Microsystems, Tokyo, Japan) and washed in a 1 × PBS buffer. Each sample collection was performed with three biological replicates.

### 4.3. RNA Isolation, cDNA Synthesis

Total RNA was extracted from the samples using the TRIzol method (Invitrogen, Carlsbad, CA, USA), and its integrity was evaluated through 1% agarose gel electrophoresis. Next, RNA was reverse transcribed into cDNA using the TransScript^®^ First-Strand cDNA Synthesis SuperMix kit (Transgen, Beijing, China), according to the manufacturer’s guidelines. Total RNA (1 μg) was isolated, stored at −80 °C, and used as a template for cDNA synthesis. The cDNA obtained was subsequently utilized in gene cloning and quantitative PCR (qPCR) experiments. The primers for gene cloning are provided in Table S1 (Supplementary Materials).

### 4.4. qPCR Analysis

Gene expression analysis was performed using the TransStart Green qPCR SuperMix Kit (TransGen Biotech, Beijing, China) on the ABI Prism 7500 Real-Time PCR System (Applied Biosystems, New York City, NY, USA), in line with the manufacturer’s guidelines. Each 20-μL reaction included 10 μL of 2× TransStart^®^ Tip Green qPCR SuperMix, 0.4 μL of passive reference dye II, 0.4 μL forward and reverse primers, 1.0 μL cDNA, and 7.8 μL of double-distilled water. The amplification protocol included an initial denaturation at 94 °C for 30 s, followed by 40 cycles of denaturation at 94 °C for 5 s, and annealing at 60 °C for 34 s, with melt curve stages at 95 °C for 15 s, 60 °C for 1 min, and 95 °C for 15 s. The *ribosomal protein L13* (*RPL13*) gene was chosen as the reference gene for normalization [38]. Each sample had three biological replicates. Data were analyzed using the 2^−ΔΔCT^ method [39]. The primers for qPCR assay are provided in Table S2 (Supplementary Materials).

### 4.5. Juvenile Hormone Analog (JHA) and 20E Treatment

To study how JHA and 20E influence ovarian development in *S. frugiperda*, methoprene (JHA, Sigma-Aldrich, St. Louis, MO, USA) was diluted to 2.5 μg/100 nL using DMSO, and 20E (Aladdin, Shanghai, China) was separately diluted to 5 μg/100 nL using the same solvent. Using a microinjector (Nano inject III, Drummond, PA, USA), 100 nL of each JHA dilution and 20E dilution were separately injected into the internode membrane of the third and fourth ventral segments of newly emerged female, unfed female *S. frugiperda* on day 0. This resulted in a final JHA concentration of 2.5 μg and a final 20E concentration of 5 μg, with an equal volume of DMSO injected as the control.

### 4.6. Determination of JH III Titer and 20E Content

The JH III titer and 20E content were determined using an enzyme-linked immunosorbent assay (ELISA) kit (Gelatins, Shanghai, China) following the product guidelines [40]. For determining JH III and 20E levels, females of *S. frugiperda* were collected on ice after RNA interference (RNAi) treatment. For each RNAi sample, three biological replicates and three technical replicates were conducted independently. Females (0.1 g) injected with RNAi were homogenized in phosphate-buffered saline (PBS, 9 mL, pH 7.2–7.4), then centrifuged (5000× *g*), and the supernatant was used for ELISA. Antibodies in microplates were captured with purified insect hormones and prepared as solid-phase antibodies. Then, the sample, standard, and HRP labeled detection antibodies were added into the microplates. After thorough washing, 3,3′, 5,5′-tetramethylbenzidine (TMB) was used for color development. Absorbance (OD value) was determined using a microplate reader at 450 nm to calculate the sample JH III titer and 20E content using the standard curve.

### 4.7. Synthesis of Double-Stranded RNA and RNA Interference

Amplification of the *Met* and *EcR* genes was performed individually, followed by cloning into the P-easy-T3 vector (Transgen, Beijing, China). Double-stranded RNAs (dsRNA) were generated using plasmid templates with a T7 high yield RNA Transcription Kit (NEB, Ipswich, New England). The specific primers used for dsRNA synthesis are shown in Table S3 (Supplementary Materials). A green fluorescent protein (GFP) was employed as the control dsRNA (dsGFP). The dsRNA concentrations were determined using a NanoPhotometer P330 spectrophotometer (Implen, München, Germany) and 1% agarose gel electrophoresis. The final concentration of the synthesized dsRNA was adjusted to 10 µg/µL.

Microinjection was used as an effective method for dsRNA delivery in insects. After being anaesthetized in ice, 1 μg dsRNA solution was administered by injection into the abdomen of newly emerging female adults (within 12 h of eclosion). The injected insects were then reared under the above conditions.

### 4.8. Monitoring of Ovarian Development and Reproductive Capacity After the Injection of dsMet and dsEcR

For the observation of ovary development, after RNAi treatment for 24 h, the ovaries were dissected and their development was observed. For fecundity assessment, all RNAi knockdown and control females were paired with a mate in a feeding cup, and daily egg counts were performed for the females until they died. For the RNAi experiment, more than 15 adult replicates were tested for each treatment.

### 4.9. Data Analysis

Statistical analysis was conducted with SPSS 22.0 and GraphPad Prism 8.0. One-way ANOVA with Tukey’s LSD test (α = 0.05) was used to assess the significance of the data. A *p*-value of less than 0.05 was considered significant (* *p* < 0.05, ** *p* < 0.01, and *** *p* < 0.001). Data are expressed as Means ± SE.

## 5. Conclusions

We utilized ELISA, qPCR, RNAi, and microdissection technologies to evaluate the impact of *Met* and *EcR* genes by JHA and 20E on the induction of ovarian development and fecundity in *S. frugiperda* females, and determined the transcription levels of JH- and 20E-related signals. Our results showed that ovary development and fecundity were promoted by JHA and 20E induction in *S. frugiperda*. Furthermore, the transcription levels of two key receptor genes of JH and 20E were markedly increased by JHA and 20E induction. In addition, the *Met* and *EcR* knockdown hindered yolk formation and egg maturation and decreased adult fecundity. Meanwhile, the gene expression of *Vg*, *kr-h1*, and *USP* was reduced after Met and EcR RNAi. Therefore, we propose that the Met and EcR genes play important roles in the reproduction of *S. frugiperda* by affecting its ovary development and egg maturation.

## Data Availability

This study includes no data deposited in external repositories.

## Supplementary Materials

The supporting information can be downloaded at: https://www.mdpi.com/article/10.3390/ijms26051914/s1.
