# Supplementary material for: Juvenile Hormone and Ecdysteroids Facilitate the Adult Reproduction Through the Methoprene-Tolerant Gene and Ecdysone Receptor Gene in the Female Spodoptera frugiperda
Source: Int J Mol Sci. 2025 Feb 23;26(5):1914. doi: 10.3390/ijms26051914 (PMC11900537; doi:10.3390/ijms26051914)
Supplement: Supplementary file 1 [file ijms-26-01914-s001.zip › ijms-3455478-supplementary.pdf]

Table S1 PCR Primers for gene amplification

Gene name	Primer name	Primer sequence（5′-3′）
Kr-h1	Kr-h1-F	GAGGAACGGGTCCATCAATGTGG
	Kr-h1-R	AATTTTCTTTTTCGTGGCGGCAG
USP	USP-F	ATTGGGTTATTCGTGATTCC
	USP-R	TCCACATATAGAGCAGAGGT
FOXO	FOXO-F	GGCGAGCTGACAGAGGTCGGTTT
	FOXO-R	TGCTGATGTGGGTGCGTGGGATG
Met	Met-F	TCTGATAATGTCCTTGTTGTCTG
	Met-R	AACTCTGTACTTTCTTTGTGGCT
EcR	EcR-F	TGCTCGGATTGTGTTGTGA
	EcR-R	GCTATTAGGGACTTCTGGTTGG

Table S2 Primers for real-time quantitative PCR

Gene name	Primer name	Primer sequence（5′-3′）
RPS13	q-RPS13-F	GCCTTAACCCTGCTTTTGCTAG
	q-RPS13-R	GCTTCGCCCTTCAATACCTTC
Kr-h1	q-Kr-h1-F	CTTTCGAACACTCCGGAAAATT
	q-Kr-h1-R	TGTGGGTGCGTAGATGGATGAC
USP	q-USP-F	CATTGGTGGATAGCGAGGGG
	q-USP-R	TCAGTGGCTAACGATGAACCTG
FOXO	q-FOXO-F	ATTACGAAACGGCGCAACA
	q-FOXO-R	CGGCGATAACTGGAAACTGC
Met	q-Met-F	GAGACTCGCCGCCAACAAG
	q-Met-R	CAAACATAACACCGCCAAACAG
EcR	q-EcR-F	TCTTCAGGCGGAGTGTAACCA
	q-EcR-R	GCCAGACATTTCTTCAACCGA
Vg	q-Vg-F	CGAAGAACCTCAAATACGAAACTGT
	q-Vg-R	TGGTGCTGGAGTGGGTAGATAA

Table S3 Primers for dsRNA synthesis

Gene name	Primer name	Primer sequence（5′-3′）
Met	dsMet-F	TAATACGACTCACTATAGGGATCCCCAAGAACTCCAAACAA
	dsMet-R	TAATACGACTCACTATAGGGGCGTAACACACAAATGACCCA
EcR	dsEcR-F	TAATACGACTCACTATAGGGGAAAATGTCAGGAGTGTCGGTT
	dsEcR-R	TAATACGACTCACTATAGGGCTGGTTGGCGGTGAGGGG
EGFP	dsEGFP-F	TAATACGACTCACTATAGGGTGAGCAAGGGCGAGGAG
	dsEGFP-R	TAATACGACTCACTATAGGGCGGCGGTCACGAACTCCAG
